# Using the Five Minute Speech Sample to assess expressed emotion in families of adolescents with anorexia nervosa

**DOI:** 10.1186/2050-2974-1-S1-O47

**Published:** 2013-11-14

**Authors:** Erica Allan, Daniel Le Grange, Susan Sawyer, Elizabeth Hughes

**Affiliations:** 1 Murdoch Childrens Research Institute, Australia; 2 Centre for Adolescent Health, Royal Children's Hospital, Australia; 3 University of Melbourne, Australia; 4 University of Chicago, Australia

## 

High Expressed Emotion (EE) has been associated with poorer treatment outcomes and increased risk for relapse in patients with eating disorders. EE refers to the attitudes, emotions and feelings expressed by a relative towards a person with an illness. Assessment of EE includes measuring levels of criticism, emotional overinvolvement (EOI), warmth and positive remarks made by a relative towards a patient.

The Structured Clinical Family Interview (SCFI) and Camberwell Family Interview (CFI) can be used to make EE ratings. However, extensive training is required to administer these interviews, and both interviews are extremely time consuming to conduct and score. The Five Minute Speech Sample (FMSS) was developed as an alternative to the SCFI and CFI and involves a relative speaking about the patient and their relationship with them for five minutes.

This presentation will describe how the FMSS is administered, provide an overview of scoring and explain the training required to score a FMSS. Extracts from FMSS recordings provided by families of patients with anorexia nervosa at The Royal Children's Hospital Eating Disorders Program will be presented. Examples of how the FMSS can be used in clinical and research settings will also be provided.

This abstract was presented in the **Anorexia Nervosa – Characteristics and Treatment** stream of the 2013 ANZAED Conference.

